# Pharmacokinetic Comparative Study of Gastrodin and Rhynchophylline after Oral Administration of Different Prescriptions of *Yizhi* Tablets in Rats by an HPLC-ESI/MS Method

**DOI:** 10.1155/2014/167253

**Published:** 2014-12-18

**Authors:** Zhaohui Ge, Yuanyuan Xie, Qionglin Liang, Yiming Wang, Guoan Luo

**Affiliations:** ^1^School of Pharmacy, Shenyang Pharmaceutical University, Shenyang 110016, China; ^2^Key Laboratory of Bioorganic Phosphorus Chemistry & Chemical Biology (Ministry of Education), Department of Chemistry, Tsinghua University, Beijing 100084, China

## Abstract

Pharmacokinetic characters of rhynchophylline (RIN), gastrodin (GAS), and gastrodigenin (*p*-hydroxybenzyl alcohol, HBA) were investigated after oral administration of different prescriptions of *Yizhi*: *Yizhi* tablets or effective parts of *tianma* (total saponins from Gastrodiae, EPT) and *gouteng* (rhynchophylla alkaloids, EPG). At different predetermined time points after administration, the concentrations of GAS, HBA, and RIN in rat plasma were determined by an HPLC-ESI/MS method, and the main pharmacokinetic parameters were investigated. The results showed that the pharmacokinetic parameters *C*_max_ and AUC_0–*∞*_ (*P* < 0.05) were dramatically different after oral administration of different prescriptions of *Yizhi*. The data indicated that the pharmacokinetic processes of GAS, HBA, and RIN in rats would interact with each other or be affected by other components in *Yizhi*. The rationality of the compatibility of *Uncaria* and *Gastrodia elata* as a classic “herb pair” has been verified from the pharmacokinetic viewpoint.

## 1. Introduction

Uncariae Ramulus Cum Uncis (*gouteng *in Chinese), the stem and hook of* Uncaria rhynchophylla* (Miq.) Jacks, was mainly used to treat hypertension, headache, vertigo, stroke, and vascular dementia in traditional Chinese medicine and Japanese Kampo medicine for centuries [1, 2]. Gastrodiae Rhizoma (*tianma* in Chinese), the root of* Gastrodia elata* Blume, is another notable Chinese medicine widely used for rheumatism, epilepsy, paralysis, hemiplegia, lumbago, headache, and vertigo in China [3]. As a classic “herb pair,” they were usually used together in some traditional Chinese medicine (TCM) formulae for the treatment of liver-yang exuberance syndrome hypertensive diseases, such as* tianma-gouteng* Yin [4].

**Figure 1:**
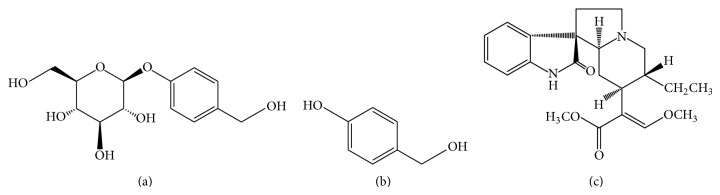
Chemical structures of three compounds: (a) GAS; (b) HBA; (c) RIN.

Alkaloids are considered the effective parts of* gouteng*, and the representative components should be rhynchophylline (Figure 1(c), RIN) and isorhynchophylline (IRN), a pair of stereoisomers at the spiro C7-position of the oxindole moiety [5]. They have exhibited potential inhibitory activity on LPS-induced excessive production of cytokines in rat primary cultured cortical microglia and N9 cells, which indicates that RIN and IRN may be effective therapeutic candidates for use in the treatment of neurodegenerative diseases [6]. Gastrodin (Figure 1(a), GAS) is the bioactive component of* tianma*, which possesses sedative and anticonvulsant properties, induces neuroprotective effects, facilitates memory consolidation and retrieval, and demonstrates antioxidant and free radial scavenging activity [7].* Yizhi* is a novel modern TCM prescription for the treatment of mild and moderate vascular dementia, which is prepared from the effective parts of* gouteng* (rhynchophylla alkaloids, EPG) and* tianma *(total saponins from Gastrodiae, EPT). RIN and GAS are considered to be the bioactive substances of* Yizhi*, with concentrations of 0.29 g/g and 2.25 mg/g in* Yizhi* tablets, respectively.

There are increasing numbers of studies on the pharmacological activity of gastrodin in the literature since the 1980s. As reported previously, the major metabolite in the blood of rats orally administered GAS was* p*-hydroxybenzyl alcohol (Figure 1(b), HBA), also named gastrodigenin, the aglycone of GAS. HBA exhibits a scavenging effect on free radicals and assists with the consolidation and retrieval of memory and is considered a bioactive component of gastrodin, together with GAS [8, 9]. Recently, some quantitative methods with relatively fine sensitivity including high performance liquid chromatography (HPLC), HPLC tandem mass spectrometry equipped with an electrospray ionization (ESI) interface, capillary micellar electrokinetic chromatography, and isotope labelling methods have been developed for the determination of GAS and/or HBA in biological samples [8–14]. The pharmacokinetic studies of GAS and/or HBA in plasma, brain, or bile were also investigated, as well as the interactions between GAS and other compatible components or extracts on the basis of pharmacokinetic parameters [8, 15, 16]. Meanwhile, the determination of RIN and its stereoisomer, IRN, in biological samples by HPLC or LC-MS methods have also been performed to understand their pharmacokinetic properties and assess their tissue distribution [17–19]. The effects on the distribution of GAS and/or RIN in rats after oral administration of the combination of* gouteng* and* tianma* have also been described previously [20]. However, studies related to the interplay of the pharmacokinetics of GAS and RIN* in vivo *have scarcely been reported to date.

To assess the pharmacokinetics of GAS, HBA, and RIN and investigate the rationality of combined applications of* gouteng* and* tianma* according to pharmacokinetic parameters, a selective and reliable HPLC-MS method for the determination of the three components in rat plasma has been developed and validated. The pharmacokinetic differences of these three components after oral administration of EPT, EPG, and* Yizhi* tablets (the compound prescription of effective parts of* tianma* and* gouteng*) to rats were also compared and discussed.

## 2. Materials and Methods

### 2.1. Materials and Reagents

Gastrodin and gastrodigenin (purity > 99%) were purchased from the National Institute for the Control of Pharmaceutical and Biological Products, Beijing, China. Rhynchophylline was purchased from the traditional Chinese medicine solid preparation manufacturing technology national engineering research centre, Jiangxi, China. The HPLC-grade methanol and acetonitrile were obtained from Merck Chemicals, Darmstadt, Germany. Triethylamine was purchased from Beijing Modern Eastern Fine Chemical, Beijing, China. Ultrapure water (18.2 MΩ) was prepared daily with a Milli-Q water purification system (Millipore, Molsheim, France) and was used in the mobile phase. Other reagents were of analytical grade.

*Yizhi* tablets,* tianma* extract (EPT, total saponins from Gastrodiae), and* gouteng* extract (EPG, rhynchophylla alkaloids) were obtained from Beijing Tech-Sky Meditech Ltd. (Beijing, China). All TCM mixtures were under careful quality control to ensure their identity throughout all of the experiments. As shown in Supplementary Table S1  in the Supplementary Material available online at http://dx.doi.org/10.1155/2014/167253, 16 components in* Yizhi* tablets were identified, and the content of RIN, GAS, and HBA was accurately quantified.

#### 2.1.1. Animals

Male Sprague-Dawley rats (weight: 220 ± 30 g) were purchased from the animal laboratory centre at Tsinghua University (Beijing, China). All animals were maintained in an environmentally controlled room under controlled temperature (22–25°C) and relative humidity (50 ± 5%) on 12 h light/dark cycles (lights on from 08:00 to 20:00). Experiments were conducted in a specific pathogen-free (SPF) grade laboratory, according to the guidelines of the Guiding Principles for the Care and Use of Laboratory Animals approved by the Committee for Animal Experiments at Tsinghua University (Beijing, China). The animals were acclimated for one week before use. Standard diet and water were provided to the rats* ad libitum*.

#### 2.1.2. Preparation of* Yizhi* Tablet,* Tianma*, and* Gouteng* Suspension

*Yizhi* tablets were prepared by mixing* Yizhi* tablet powder (9 g) in 10 mL 5% CMC-Na with a magnetic stirring apparatus. The concentration of effective parts of* tianma *(EPT, total saponins from Gastrodiae) and* gouteng* (EPG, rhynchophylla alkaloids) was equivalent to that in a* Yizhi *tablet. The three different suspensions were shacked uniformly before administration.

### 2.2. Animal Treatment

The rats were randomly assigned to one of three groups (1–3), representing the groups administered with EPT, EPG, and* Yizhi*, respectively. Each group contained six rats. The rats fasted overnight with free access to water before dose administration. After a single dose was administered by oral gavage, blood samples (0.5 mL) were collected in heparinized tubes via the orbital vein at 0 min, 10 min, 20 min, 30 min, 45 min, 1 h, 2 h, 4 h, 6 h, 8 h, and 12 h. All blood samples were centrifuged at 3000 rpm for 15 min to collect plasma. The plasma samples obtained were stored at −20°C until analysis. Along with the plasma samples, quality control (QC) samples were distributed among calibrators and unknown samples in the analytical run. All of the blank samples used as controls were prepared by the same method as drug-containing samples.

### 2.3. Blood Sample Preparation

To 200 *μ*L of thawed plasma, 25 *μ*L aqueous ammonia and 800 *μ*L ether were added. The supernatant was separated and dried under nitrogen gas at 37°C after vortexing for 1 min and centrifuging at 10,000 g for 15 min at 4°C. The residues were reconstituted in 50 *μ*L of methanol and centrifuged at 15,000 g for 15 min. A 20 *μ*L aliquot of the supernatant was injected into the HPLC-MS system for analysis of RIN. To the residue, 800 *μ*L methanol was added. The supernatant was separated and evaporated to dryness under nitrogen at room temperature after being vortexed for 3 min and centrifuged at 10,000 g for 15 min. The residue was reconstituted in 50 *μ*L of the initial mobile phase and centrifuged at 15,000 g for 15 min. 20 *μ*L aliquot of the supernatant was injected into the HPLC-MS system for analysis of GAS and HBA.

### 2.4. Determination of GAS, HBA, and RHY in Plasma

#### 2.4.1. Apparatus

The HPLC-MS system consisted of an Agilent 1200 series HPLC system (Agilent Technologies Corp., Santa Clara, CA, USA) equipped with a binary solvent delivery system and autosampler, in tandem with an Agilent G1969A LC/MSD TOF (time-of-flight) mass spectrometer (Agilent Technologies Corp., Santa Clara, CA, USA) equipped with an electrospray ionization (ESI) interface.

#### 2.4.2. Chromatographic Conditions

The HPLC separation was performed on an Agilent HC-C^18^ column (Agilent Technologies Corp., Palo Alto, CA, USA; 250 mm × 4.6 mm, 5 *μ*m particle size) at 35°C. For the analysis of GAS and HBA, the mobile phase comprised a gradient system of methanol (solution A) and water containing 0.01 M triethylamine (pH 8.0, solution B) at a flow rate of 1 mL/min as follows: a linear gradient of the mixture of solutions (A : B) from 10 : 90 (v/v) to 30 : 70 (v/v) for 20 min. For the analysis of RIN, an isocratic elution was performed over 30 min with the mobile phase comprising methanol-0.01 M aqueous triethylamine (37 : 63, v/v) at the a flow rate of 1 mL/min. The equilibration time was 15 min, and the flow rate was reduced to 0.375 mL/min prior to MA detection using a T-split. TOF-MS analysis was performed in negative ion mode (ESI^−^) for detecting GAS, HBA, and RIN. The operation parameters were the following: drying gas (nitrogen) flow rate 9 L/min, drying gas temperature 350°C; nebulizing gas (nitrogen), 35 psi; fragmentation voltage 175 V; skimmer voltage 60 V; octupole DC-1 37 V; capillary voltage 3500 V. Data files were acquired in continuum (profile) mode and spectra were stored from* m/z* 50 to 700. Accurate mass measurements of each peek from the total ion chromatograms were obtained by means of an automated delivery system using a dual-nebulizer electrospray source that introduces the flow from the outlet of the chromatograph together with a low flow of the reference solution (reference solution A, Agilent Technologies). The internal reference mass* m/z* = 112.985599 was chosen in the negative mode. MassHunter Workstation software (Agilent Technologies, Santa Clara, CA, USA) was used for data acquisition.

#### 2.4.3. Preparation of Standard Solution

Primary stock solutions of GAS, HBA, and RIN were prepared at a final concentration of 1.0 mg/mL, by dissolving accurately weighed reference compounds in methanol. To prepare the standards for calibration, stock solutions were serially diluted with methanol to obtain the desired concentrations. All of the solutions were stored at 4°C and were brought to room temperature before use. Calibration standards of GAS, HBA, and RIN were prepared by spiking 100 *μ*L of the standard solutions in 200 *μ*L pooled blank plasma samples, and the resulting nominal plasma concentrations were within the range of 1 to 250 *μ*g/mL for GAS, 1 to 100 *μ*g/mL for HBA, and 0.25 to 50 *μ*g/mL for RIN, respectively. For each validation and assay run, the calibration curve standards were prepared fresh from the working solutions. Quality control (QC) samples were prepared by individually spiking control rat plasma at three concentration levels: low, medium, and high. All samples were stored at −20°C until analysis.

### 2.5. Validation Procedures

#### 2.5.1. Selectivity

Selectivity of the method was evaluated by analysing plasma samples collected from six different rats to investigate potential interferences for analytes using the proposed extraction procedure and HPLC conditions.

#### 2.5.2. Calibration Curves and Linearity

Calibration curves were prepared by spiking pooled control rat plasma with standard working solutions (100 *μ*L each) to produce the calibration curve points. An external standard method was used for the calculation of plasma concentrations of determined components. The linearity was detected by calculating the correlation coefficient (*r*) of the curves by means of weighted (1/*X* ^2^) least squares linear regression. All calibration curves of the three determined components were constructed prior to the experiments with a correlation value of at least 0.99.

#### 2.5.3. Precision and Accuracy of the Assay

Accuracy and precision were assessed by determination of QC samples at three concentrations (15, 100, and 250 *μ*g/mL for GAS and 2, 10, and 50 *μ*g/mL for RIN and HBA) in six replicates on three validation days. Precision was expressed by relative standard deviation (RSD) and accuracy by relative error (RE). The intra- and interday precisions were required to be less than 15%, and the accuracy was required to be within ±15%. The lower limit of quantitation (LLOQ) was determined quantitatively by an analytical method with a precision of no worse than 20% and an accuracy within ±20%. LLOQ was evaluated by analysing samples prepared in six replicates on three separate days.

#### 2.5.4. Recovery

Extraction recovery, evaluated in three replicates of each QC sample (10, 50, and 100 *μ*g/mL for GAS and HBA and 1, 5, and 10 *μ*g/mL for RIN), was determined by comparing the peak areas of extracted plasma (prespiked) standard QC samples to those of postspiked standards at equivalent concentrations.

#### 2.5.5. Stability

The stability experiments on GAS, HBA, and RIN were performed at three concentrations of the QC samples, using three replicates at each concentration (10, 50, and 100 *μ*g/mL for GAS and HBA and 1, 5, and 10 *μ*g/mL for RIN). Short-term stability was determined after exposure of the spiked samples at room temperature for 24 h. The freeze/thaw stability was evaluated after three complete freeze/thaw cycles (−80°C) on consecutive days. Long-term stability was assessed after storage of the standard spiked plasma samples at −80°C for 20 days. Postextraction stability was determined after the extracted samples had been stored in the autosampler pending analysis at the temperature of the autoinjector (4°C) for 24 h. The analytes were considered stable in the biological matrix when 85–115% (RE) of the initial concentration was found.

### 2.6. Pharmacokinetic Analysis

*Yizhi *tablets, EPT, and EPG were separately suspended in 0.5% carboxymethylcellulose solution and administered orally to six conscious rats at doses of 9 g/kg for* Yizhi* tablets, 5.4 g/kg for EPT, and 3.6 g/kg for EPG, respectively. Blood samples were collected from the retroorbital plexus of rats under light anaesthesia with ether. Blood samples were collected in microfuge tubes containing heparin as an anticoagulant at 0 min, 10 min, 20 min, 30 min, 45 min, 1 h, 2 h, 4 h, 6 h, 8 h, and 12 h after treatment. Plasma was harvested by centrifuging blood samples at 3500 ×g for 10 min and stored at −80°C until analysis. The concentrations of GAS, HBA, and RIN in plasma were determined as described above. QC samples were distributed among calibrators and samples in the analytical run to adjust the error.

### 2.7. Data Analysis

The pharmacokinetic parameters, such as area under the plasma concentration-time curve (AUC), maximum plasma concentration (*C*_max⁡_), corresponding time (*T*_max⁡_) and half-life (*T*_1/2_), plasma clearance (CL), mean residence time (MRT), and initial plasma concentration (*C*_0_) for orally administered dose, were performed on each individual set using the software of Kinetica (Version 5.0, Thermo Electron Corp., MA, USA) by the noncompartmental model. Data were presented as the mean ± standard deviation (S.D.). Comparisons of the pharmacokinetic data were performed by one-way analysis of variance (ANOVA), and the statistically significant difference was set at a value of *P* < 0.05 (SPSS statistical software package, Version 17.0, SPSS Inc., Chicago, IL, USA).

## 3. Results and Discussion

### 3.1. Optimization of Sample Preparation

To avoid the occurrences of ionization suppression and detection interference during the HPLC-ESI-MS analysis, the preliminary plasma sample extraction was optimized in this study. Due to the polarity of RIN, medium QC samples were liquid-liquid extracted with different organic solvents, including ether, chloroform, and ethyl acetate. Ether was finally chosen as the extraction reagent due to its good intermiscibility with the analytes and extraction recoveries close to 90%. Subsequently, the aqueous phase after liquid-liquid extraction was deproteinized for the determination of HBA and GAS with different extraction solvents within different proportions, including acetonitrile, methanol, perchloric acid, sodium hydroxide, ethyl acetate, and ether. Quintuple methanol in volume of the plasma extraction offered a very pleasant result in that the extraction recoveries were close to 90% and offered the least interference with endogenous compounds.

### 3.2. Optimization of Chromatography and Mass Spectrometry Conditions

Due to the existence of positional isomers; varied types, polarities, and levels of analytes; complex matrices of plasma samples; and the plausible interference of two drugs* in vivo*, the influence of many parameters was studied to achieve good separation and high sensitivity. These parameters included the analysing column, flow rate, and pH value of mobile phase, ionization polarity, and fragmentation voltage. The tested HPLC columns were all packed with C_18_, including Phenomenex Luna C_18_ (250 mm × 4.6 mm, 5 *μ*m, Phenomenex. Inc., Torrance, CA, USA), Agilent TC-C_18_ (250 mm × 4.6 mm, 5 *μ*m, Agilent Technologies, Santa Clara, CA, USA), and Agilent HC-C_18_ (250 mm × 4.6 mm, 5 *μ*m, Agilent Technologies, Santa Clara, CA, USA) columns. Finally, an Agilent HC-C_18_ column was chosen for its better separation of the analytes than the other two columns. As reported before, Agilent HC-C_18_ columns showed favorable performance in separating nonpolar compounds, particularly the ones containing basic groups, and were more applicable to the components without sufficient retention in common C_18_ columns according to the surface properties of its packing material.

The main component of mobile phase in reverse-phase chromatography is water. Methanol was used as the organic component, which produced both acceptable peak shape and stable, sensitive mass spectral performance of all of the analytes. The addition of ammonia (0.1%, 0.2%) or triethylamine (0.01 M, 0.02 M, or 0.03 M) to the mobile phase improved the resolution and sensitivity by suppressing the ionization of the alkaloid components. To achieve symmetrical peak shapes and high sensitivity, a mobile phase consisting of methanol and water (0.01 M triethylamine, pH adjusted to 8.0 with glacial acetic acid) was used in the experiment.

**Figure 2:**
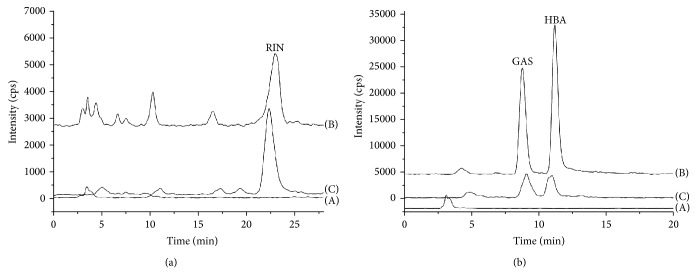
Representative XIC chromatograms of LC-MS. (a) XIC chromatograms of RIN in (A) blank plasma, (B) blank plasma spiked with standard RIN, and (C) plasma after oral administration of drugs. (b) XIC chromatograms of GAS and HBA in (A) blank plasma, (B) blank plasma spiked with standard GAS and HBA, and (C) plasma after oral administration of drugs.

As aforementioned, some quantitative methods with relatively fine sensitivity to HPLC, HPLC-ESI/MS, and CE have been developed for the determination of GAS, HBA, and/or RIN in biological samples. Although a DAD showed better performance than an MS detector in terms of stability and repeatability, considering the challenges of sample complexity and detector sensitivity, an MS detector was chosen for the determination. Multiple extracted ion chromatograms (XICs), coming from total ion chromatograms by using Analyst QS software, were used for compound identification and quantification. The multiple XICs of a representative blank sample spiked with standards and internal standards are displayed in Figures 2(a) and 2(b). The main characteristic ions in positive and negative modes are shown in Table 1. Ions for quantification were selected mainly by comparing the signal/noise ratios in two detecting modes at the same concentration, and the negative mode was finally chosen.

### 3.3. Method Validation

#### 3.3.1. Assay Selectivity

Selectivity was assessed by comparing the chromatograms of six separate batches of control rat plasma (blank plasmas) with corresponding spiked plasma samples. Under the current optimised HPLC-MS conditions, GAS, HBA, and RIN were all baseline separated. Blank plasma yielded relatively clean chromatograms without significant peaks interfering with the three analytes. As shown in Figure 2, the typical chromatogram obtained from dosed rats displayed peaks not present in the chromatogram of a blank sample. The representative peaks had the same* m/z* values as the standard samples. No interference peaks were detected for GAS, HBA, and RIN from the plasma. The retention time of approximately 8.75 min for GAS, 11.25 min for HBA, and 23.6 min for RIN lags far behind the dead time and the retention time of the endogenous substances in plasma, contributing to the avoidance of main interferences.

#### 3.3.2. Calibration Curve

The plasma calibration curve was constructed using seven calibration standards (1–250 *μ*g/mL for GAS, 1–100 *μ*g/mL for HBA, and 0.5–50 *μ*g/mL for RIN, which spanned the concentrations typically found in a rat's plasma after administration of* Yizhi* tablets, EPT, and EPG in the pharmacokinetic study. The calibration standard curve had a reliable reproducibility as determined by the best fit of peak area versus concentration and fit to *Y* = *aX* + *b* using weighing factor (1/*X* ^2^). The correlation coefficient was found to be ≥0.990, indicating good linearity. The lowest concentration with an RSD <20% was considered as the LLOQ and those for GAS, HBA, and RIN were found to be 500, 500, and 380 ng/mL, respectively (Table 2).

#### 3.3.3. Precision and Accuracy

The precision of the method was determined by calculating RSD for QC samples at three concentrations over three validation days. The intraday precision was 8.23% or less, and the interday precision was 8.64% or less at each QC sample concentration. The accuracy of the method, expressed in terms of RE, ranged from −7.54% to −2.68% at the three QC sample concentrations. The above results (Table 3) demonstrated that the values were within the acceptable range and the method was both accurate and precise.

#### 3.3.4. Extraction Recovery

Mean extraction recoveries of GAS, HBA, and RIN ranged from 87.7% to 100.0%, 87.9% to 91.3%, and 94.0% to 99.2%, respectively, as shown in Table 4.

#### 3.3.5. Stability

The stability results showed that GAS, HBA, and RIN spiked into rat plasma were stable when kept at room temperature (22–25°C) for 24 h, at −80°C for 20 days, and during three freeze/thaw cycles, as shown in Table 5. The RSDs of each compound's stability were all lower than 10% and no significant degradation was observed.

### 3.4. Plasma Pharmacokinetics Study

**Figure 3:**
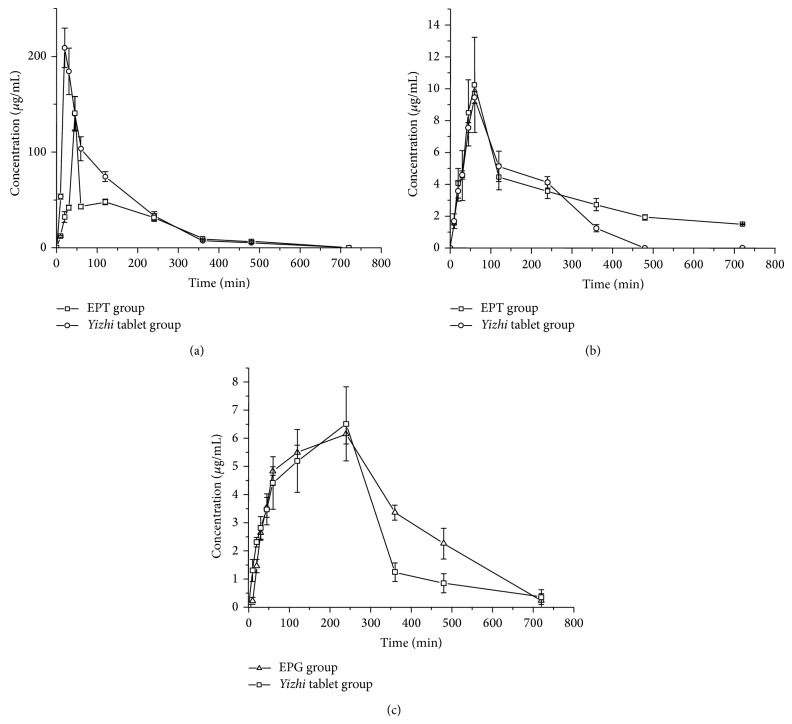
Mean plasma concentration-time profiles in rat plasma after oral administration. Each point represents the mean ± SD. ((a) GAS, (b) HBA, and (c) RIN).

The validated HPLC-MS method was used to quantitatively determine plasma concentrations of GAS, HBA, and RIN after an oral administration of* Yizhi* tablets, EPT, or EPG to rats. Their mean plasma concentration versus time profiles is shown in Figure 3. The corresponding pharmacokinetic parameters generated by fitting plasma concentration profiles to a noncompartmental model are summarized in Table 6, which were expressed as the mean ± SEM (*n* = 6). The pharmacokinetic profiles of combined administration were found to be significantly different from those administered individually.

We found that GAS in rat plasma reached the *C*_max⁡_ at 21.67 min and was rapidly eliminated from the plasma. The *T*_max⁡_ value of the single administration group (EPT group) in our studies was found to be similar to that reported [7, 15]. *C*_max⁡_ and AUC_0–*t*_ were 140.72 *μ*g/mL and 230.27 *μ*g h/mL, respectively. The absorption rate of GAS was obviously slowed down to 45 min, and MRT and AUC were increased by 1.32-fold and 1.56-time, respectively, when coadministered with* Yizhi* tablet. Similar results have also been reported when* tianma* was used in combination with* chuanxiong* (Ligusticum chuanxiong Hort., Umbelliferae) [7]. The plasma concentration of GAS in the* Yizhi* tablet group showed two successive maximum concentrations, which occurred individually at 45 min and 120 min following treatment with* Yizhi* tablets. In contrast, a plateau in the plasma concentration-time profile of GAS in the plasma of the EPT group appeared during the same time period. This phenomenon indicated that the combination of EPT and EPG in* Yizhi* tablets might cause the enterohepatic circulation of GAS. The major metabolite of GAS detected in the plasma of rats orally given EPT or* Yizhi* tablets was gastrodigenin (p-hydroxybenzyl alcohol), the aglycone of GAS, which is consistent with the results previously reported. Compared with EPT administered alone, EPT coadministered with EPG (*Yizhi* tablet) has markedly increased AUC and longer MRT (*P* < 0.01), as well as higher *C*_max⁡_, which account for the higher bioavailability of HBA in the* Yizhi* tablet group.

RIN in rat plasma reached *C*_max⁡_ at 220 min and was slowly eliminated from the plasma. *C*_max⁡_ and AUC_0–*t*_ were 6.17 *μ*g/mL and 40.24 *μ*g h/mL, respectively. The absorption rate of RIN was obviously slowed down to 240 min, and MRT and AUC were decreased to 223.06 min and 29.72 *μ*g h/mL, respectively, when coadministered with* Yizhi* tablet intragastric administration. The delay of *T*_max⁡_ may prolong the effective time of the drug and enhance the medication effect. The present pharmacokinetic study of RIN provides evidence of a 20 min delay, indicating that absorption of RIN could be slower when administered with* Yizhi* tablets than with EPG alone. The mechanisms accounting for the decreased absorption of RIN are still not clear, as well as the effect of this decrease on the efficacy and safety of formula, which needs more research on the precise mechanism of the mutual effect on absorption between GAS and RIN.

In TCMs, pharmacokinetic parameter differences between single herbal and compound herbal prescriptions are common. Zheng et al. investigated the influence of compatibility of the pharmacokinetics of the active ingredient in Gastrodin by comparative evaluations of the pharmacokinetics of the Da Chuan Xiong Decoction Compound Preparation (DCXDCP) with various combinations of its constituent herbs in plasma after oral administration. The results indicated that the compatibility effects of other ingredients present in DCXDCP could affect the pharmacokinetics of the prescription [7]. These differences in pharmacokinetic properties between these two research results note the importance of investigating the pharmacological characteristics of the compound herbal prescriptions used in clinical situations.

## 4. Conclusion

An HPLC method with MS detection was developed and applied to the determination of GAS, HBA, and RIN in the plasma of rats dosed with EPT, EPG, and their compatibility proportion extract suspensions, and the pharmacokinetic parameters were compared. The pharmacokinetic results obtained may be useful for further study of the mechanism of* Yizhi* tablets. The results of this study showed that the pharmacokinetic parameters of GAS, HBA, and RIN were dramatically different after oral administration of EPT, EPG, and* Yizhi* tablets, the combinations of its constituent herbs.* Gouteng* had some retarding influence on the absorption, distribution, and elimination of GAS* in vivo* because the pharmacokinetic parameters of GAS were significantly different following oral administration of EPT and the different combinations of its constituent herbs. EPT has the same effect on RIN as well. The rationale of the compatibility of* Uncaria* and* Gastrodia elata* as a classic “herb pair” has been verified from the point of pharmacokinetics.

## Supplementary Material

A reliable and powerful analytical method by using the integrative strategy of simultaneous qualification and quantification of multi-component for the comprehensive quality evaluation of *Yizhi* Tablet has been established. TCM fingerprinting together with the identification of unknown components on-line by HPLC coupled with time-of-flight (TOF) tandem mass spectrometry and HPLC-ESI multi-stage tandem ion-trap mass spectrometry (IT-MS^n^) method. The structure information of 14 components in *Yizhi* Tablet were identified.

## Figures and Tables

**Table 1:** HPLC-ESI-MS accurate mass measurement of the three compounds.

Compound	Retention time	Ion	Elemental compositions	Theoretical	Experimental	DBE	Error	
	(min)			*m*/*z*	*m*/*z*		mDa	ppm
RIN	23.56	[M-H]^−^	C_22_H_27_N_2_O_4_	383.1970	383.2024	10.5	2.6373	13.876
GAS	8.75	[M+CH_3_COO]^−^	C_15_H_21_O_9_	345.1185	345.1212	5.5	1.8424	5.3384
HBA	11.25	[M-H-H_2_O]^−^	C_7_H_5_O	105.0340	105.0339	5.5	−0.1398	−1.3313

**Table 2:** Regression equations, correlation coefficients (*r*), linearity ranges, and lower limit of quantitation (LLOQ) for GAS, HBA, and RIN in plasma.

Marker compounds	Calibrations	*r*	Linear range (*μ*g/mL)	LLOQ (*μ*g/mL)
GAS	*y* = 19689.46*x* − 89461.47	0.9907	1–250	0.50
HBA	*y* = 46483.29*x* − 51323.08	0.9994	1–100	0.50
RIN	*y* = 36880.5*x* + 3717.92	0.9916	0.25–50	0.38

**Table 3:** Precision and accuracy of HPLC-MS method in rat plasma (*n* = 6).

Marker compounds	Concentration (*μ*g/mL)		RSD (%)		RE (%)
	Added	Found	Intraday	Interday	
GAS	15	13.7 ± 0.3	4.49	8.23	4.5
	100	102.7 ± 2.3	4.57	3.78	4.6
	250	242.9 ± 2.0	1.64	6.21	1.6

HBA	2	1.9 ± 0.06	6.25	6.70	6.2
	10	9.3 ± 0.4	8.62	2.80	8.6
	50	46.2 ± 1.9	8.32	4.51	8.3

RIN	2	1.9 ± 0.04	4.02	4.08	4.0
	10	9.8 ± 0.4	8.64	7.43	8.6
	50	48.3 ± 1.6	6.46	8.47	6.5

**Table 4:** Recovery of GAS, HBA, and RIN in rat plasma by HPLC-MS method (*n* = 6).

Marker compounds	Concentration	Recovery (%)	RSD (%)
	(*μ*g/mL)	Intraday	
GAS	100	93.73	2.91
	50	92.68	0.64
	10	94.35	4.39

HBA	100	90.78	4.53
	50	94.35	4.27
	10	97.08	1.84

RIN	10	92.38	6.93
	5	97.32	5.08
	1	90.39	8.40

**Table 5:** Stability of GAS, HBA, and RIN in rat plasma by HPLC-MS method (*n* = 3).

Marker compounds	Concentration (*μ*g/mL)	Stability (RE, %)		
		−80°C/20 days	Freeze-thaw (3 cycles)	20°C/24 h
GAS	100	8.01	8.26	3.93
	50	6.95	4.12	3.17
	10	4.38	5.36	9.35

HBA	100	7.15	7.03	3.17
	50	5.63	4.65	2.65
	10	4.58	2.65	4.69

RIN	10	5.49	4.56	9.35
	5	8.04	8.08	7.65
	1	6.88	7.18	8.63

**Table 6:** Pharmacokinetic parameters for metabolites in rat plasma after oral administration of *Yizhi* tablets, EPT, or EPG (*n* = 6).

Parameters	Unit	*Yizhi* tablet group			EPT group			EPG group		
		GAS	HBA	RIN	GAS	HBA	RIN	GAS	HBA	RIN
*T*_max⁡_	min	45 ± 0.0	55 ± 12.25	240 ± 0.0	21.67 ± 4.08	60 ± 0.0				220 ± 48.99
*C*_max⁡_	*μ*g mL^−1^	140.72 ± 19.05	10.45 ± 3.02	6.50 ± 1.44	188.45 ± 19.33	9.12 ± 0.61				6.17 ± 0.38
AUC_(0–*t*)_	*μ*g min mL^−1^	13816.35 ± 445.39	2267.27 ± 158.92	1783.54 ± 411.33	21549.67 ± 2302.7	1656.31 ± 77.34				2414.50 ± 157.78
AUC_(0–*∞*)_	*μ*g min mL^−1^	14910.3 ± 509.41	3129.68 ± 138.9	1918.26 ± 540.62	22048.87 ± 2408.02	2080.71 ± 214.3				2604.81 ± 266.89
MRT_(0–*t*)_	min	161.69 ± 4.69	266.67 ± 14.7	223.06 ± 17.10	121.74 ± 2.03	154.12 ± 4.6				286.94 ± 16.02
MRT_(0–*∞*)_	min	197.31 ± 5.44	553.26 ± 71.54	275.13 ± 68.43	132.43 ± 3.73	238.75 ± 39.79				335.55 ± 44.75
CL/F	mL min^−1^ kg^−1^	3.50 ± 0.10	—	2.18 ± 0.46	2.32 ± 0.25	—				1.55 ± 0.13

## References

[1] Bensky D., Gamble A., Kaptchuk T. (1999). *Chinese Herbal Medicine: Material Medica*.

[2] Shimada Y., Terasawa K., Yamamoto T., Maruyama I., Saitoh Y., Kanaki E. (1994). A well-controlled study of Choto-san and placebo in the treatment of vascular dementia. *Journal of Traditional Medicine*.

[3] Committee of Pharmacopoeia of P.R. China (2010). *Chinese Pharmacopoeia. Part I*.

[4] Ma Z. W., Liu Y. F., Ji C., Cheng W. P. (2011). A review on treating Parkinson disease in TCM. *Clinical Journal of Chinese Medicine*.

[5] Yuan D., Ma B., Yang J.-Y., Xie Y.-Y., Wang L., Zhang L.-J., Kano Y., Wu C.-F. (2009). Anti-inflammatory effects of rhynchophylline and isorhynchophylline in mouse N9 microglial cells and the molecular mechanism. *International Immunopharmacology*.

[6] Yuan D., Ma B., Wu C., Yang J., Zhang L., Liu S., Wu L., Kano Y. (2008). Alkaloids from leaves of *Uncaria rhychophylla* and their inhibitory activity on NO production in lipopolysaccharide-activated microglia. *Journal of Natural Products*.

[7] Zheng Q., Yue P.-F., Wu B., Hu P.-Y., Wu Z.-F., Yang M. (2011). Pharmacokinetics comparative study of a novel Chinese traditional herbal formula and its compatibility. *Journal of Ethnopharmacology*.

[8] Li L. L., Zhang Z. R., Gong T., He L. L., Deng L. (2006). Simultaneous determination of Gastrodin and Ligustrazine hydrochloride in dog plasma by gradient high-performance liquid chromatography. *Journal of Pharmaceutical and Biomedical Analysis*.

[9] Zhang W., Sheng Y. X., Zhang J. L. (2008). Determination and pharmacokinetics of gastrodin and p-hydroxybenzylalcohol after oral administration of Gastrodia elata Bl. extract in rats by HPLC-ESI/MS method. *Phytomedicine*.

[10] Ju X. H., Shi Y., Liu N., Guo D. M., Cui X. (2010). Determination and pharmacokinetics of gastrodin in human plasma by HPLC coupled with photodiode array detector. *Journal of Chromatography B: Analytical Technologies in the Biomedical and Life Sciences*.

[11] Lin L.-C., Chen Y.-F., Lee W.-C., Wu Y.-T., Tsai T.-H. (2008). Pharmacokinetics of gastrodin and its metabolite p-hydroxybenzyl alcohol in rat blood, brain and bile by microdialysis coupled to LC-MS/MS. *Journal of Pharmaceutical and Biomedical Analysis*.

[12] Liu K. X., Han G. Z., Chang Y. L., Su C. Y., Tang N. Y., Chen Y. R. (1987). Simultaneous determination of gastrodin and its metabolite by HPLC. *Biomedical Chromatography*.

[13] Lu G. W., Zou Y. J., Mo Q. Z., Huang J., Chu D. Q., Ye D. Y. (1986). Circadian rhythm of [3H] gastrodin pharmacokinetics in rats. *Acta Pharmacologica Sinica*.

[14] Wang Q., Chen G., Zeng S. (2008). Distribution and metabolism of gastrodin in rat brain. *Journal of Pharmaceutical and Biomedical Analysis*.

[15] Jiang L., Dai J., Huang Z., Du Q., Lin J., Wang Y. (2013). Simultaneous determination of gastrodin and puerarin in rat plasma by HPLC and the application to their interaction on pharmacokinetics. *Journal of Chromatography B: Analytical Technologies in the Biomedical and Life Sciences*.

[16] Kushida H., Fukutake M., Tabuchi M., Katsuhara T., Nishimura H., Ikarashi Y., Kanitani M., Kase Y. (2013). Simultaneous quantitative analyses of indole and oxindole alkaloids of Uncaria Hook in rat plasma and brain after oral administration of the traditional Japanese medicine Yokukansan using high-performance liquid chromatography with tandem mass spectrometry. *Biomedical Chromatography*.

[17] Ma B., Sun G. B., Xu H. B., Li M., Yang Z. H., Sun X. B. (2012). Rhynchophylline in rat blood by high-performance liquid chromatography-coupled microdialysis. *Journal of Medicinal Plants Research*.

[18] Wang W., Ma C.-M., Hattori M. (2010). Metabolism and pharmacokinetics of rhynchophylline in rats. *Biological and Pharmaceutical Bulletin*.

[19] Cai J. Z., Lin C. L., Ma J. S., Hu L. F., Lin G. Y., Wang X. Q. (2013). Determination of rhynchophylline in rat plasma by liquid chromatography mass spectrometry and its application. *Journal of Chromatographic Science*.

[20] Chen Z. Y., Wang X., Li X. Q., Wang X. Y., Zhang W. G. (2012). Distribution of isorhynchophyline in liver tissues before and after compatibility with gastrodin and isorhynchophylline in rats. *Zhongguo Shiyan Fangjixue Zazhi*.

