# Supplementary material for: Pharmacokinetic Comparative Study of Gastrodin and Rhynchophylline after Oral Administration of Different Prescriptions of Yizhi Tablets in Rats by an HPLC-ESI/MS Method
Source: Evid Based Complement Alternat Med. 2014 Dec 18;2014:167253. doi: 10.1155/2014/167253 (PMC4281461; doi:10.1155/2014/167253)
Supplement: Supplementary file 1 — A reliable and powerful analytical method by using the integrative strategy of simultaneous qualification and quantification of multi-component for the comprehensive quality evaluation of Yizhi Tablet has been established. TCM fingerprinting together with the identification of unknown components on-line by HPLC coupled with time-of-flight (TOF) tandem mass spectrometry and HPLC-ESI multi-stage tandem ion-trap mass spectrometry (IT-MSn) method. The structure information of 14 components in Yizhi Tablet were identified. [file 167253.f1.pdf]

Peak no	t_R(min)	Selected ion	Formula	Measured mass(m/z)	Calculated mass(m/z)	Error		DBE	Compound
						mDa	ppm		
1	10.325	$[M-H]^-$	$C_{13}H_{17}O_7$	285.0961	285.0974	-1.3281	-4.6586	5.5	Gastrodin*
2	16.26	$[M-H]^-$	$C_{10}H_8O_4$	191.0348	191.0344	0.3661	1.9167	7.5	Scopoletin
3	21.959	$[M-H]^-$	$C_{15}H_{14}O_6$	289.0722	289.0712	0.9866	3.4131	9.5	Epicatechin
4	37.917	$[M-H]^-$	$C_{27}H_{34}N_2O_9$	529.2189	529.2186	0.2939	0.5553	12.5	Vincoside/Strictosidine
5	48.175	$[M-H]^-$	$C_{27}H_{34}N_2O_9$	529.2184	529.2186	-0.2060	-0.3894	12.5	Vincoside/Strictosidine
6	49.152	$[M-H]^-$	$C_{26}H_{30}N_2O_8$	497.1918	497.1923	-0.5912	-1.1892	13.5	Vincoside Lactam/Strictosamide
7	46.004	$[M-H]^-$	$C_{26}H_{30}N_2O_8$	497.1914	497.1923	-0.9912	-1.9937	13.5	Vincoside Lactam/Strictosamide
8	50.039	$[M-H]^-$	$C_{22}H_{26}N_2O_4$	381.1817	381.1814	0.2674	0.7015	11.5	(Iso)Corynoxeine
9	52.011	$[M-H]^-$	$C_{22}H_{26}N_2O_4$	381.1805	381.1814	-0.9325	-2.4465	11.5	(Iso)Corynoxeine
10	53.151	$[M-H]^-$	$C_{22}H_{28}N_2O_4$	383.1970	383.1970	-0.0826	-0.2157	10.5	Rhynchophylline*
11	47.524	$[M-H]^-$	$C_{27}H_{34}N_2O_{10}$	545.2134	545.2135	-0.1207	-0.2214	12.5	Dihydrocadambine

12	41.734	$[M-H]^-$	$C_{21}H_{26}N_2O_4$	369.1818	369.1814	0.3674	0.9952	10.5	(Iso)Rhynchophyllic Acid
13	42.494	$[M-H]^-$	$C_{21}H_{26}N_2O_4$	369.1817	369.1814	0.2674	0.7243	10.5	(Iso)Rhynchophyllic Acid
14	51.142	$[M-H]^-$	$C_{22}H_{28}N_2O_4$	383.1973	383.1970	0.2173	0.5671	10.5	Corynoxine
15	41.427	$[M-H]^-$	$C_{21}H_{24}N_2O_4$	367.1663	367.1657	0.5175	1.4094	11.5	(Iso)Pteropodine
16	42.874	$[M-H]^-$	$C_{21}H_{24}N_2O_4$	367.1656	367.1657	-0.1824	-0.4970	11.5	(Iso)Pteropodine

Supplementary

Tables S1 Main components identified in Yizhi Tablet by HPLC-MS

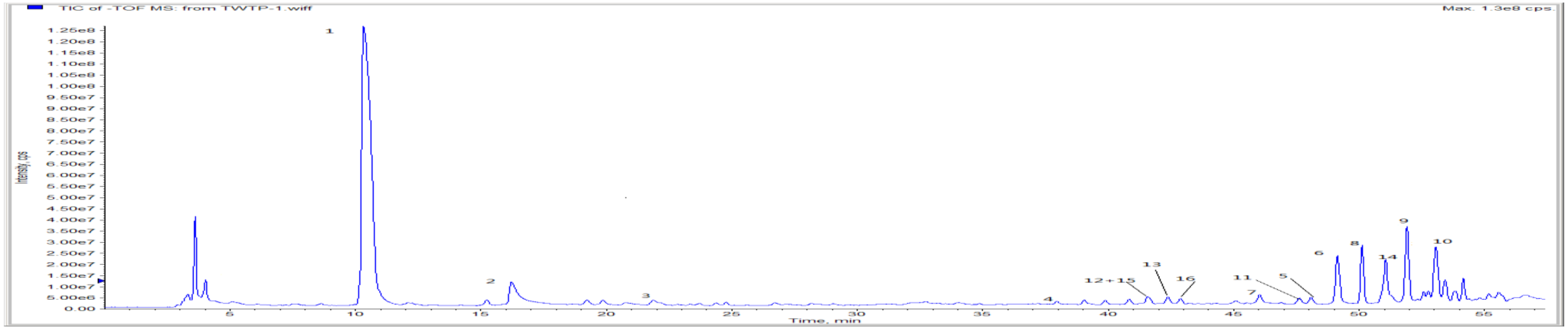

Fig.S1 Total ion chromatogram (TIC) profile of Yizhi Tablet (negative ion mode). The number of peaks marked in Fig.S1 is corresponding to Table S1.
